# IP-ConTex: detail-consistent texture generation with image prompt

**DOI:** 10.1186/s42492-026-00232-2

**Published:** 2026-09-29

**Authors:** Lei Wang, Jieqing Feng

**Affiliations:** State Key Lab of CAD&CG, Zhejiang University, Hangzhou, Zhejiang 310058 China

**Keywords:** Diffusion models, Texture generation, Texture transfer

## Abstract

Textures are critical for enhancing the visual fidelity and diversity of three-dimensional (3D) models. Recently, generative models have significantly advanced texture generation. However, fine-grained control of the generation process remains challenging. Hence, we propose IP-ConTex, which is a novel image-guided texture-generation method that introduces appearance control into the diffusion process to ensure detail-consistent results. First, synchronized multiview diffusion is employed to maintain structural and layout consistency across multiple views. Subsequently, a new appearance-control module is designed for the pretrained diffusion model. By leveraging cross-attention control, self-attention control, diffusion feature synchronization, and color adjustment, appearance information from the reference image is effectively extracted and injected into the texture-generation process. Experimental results demonstrate that IP-ConTex successfully transfers appearance details to 3D geometries without fine-tuning or optimization, thus achieving high-quality and detail-consistent texture generation.

## Introduction

Owing to rapid digitalization, fields such as digital twins, architectural design, films, and games increasingly use three-dimensional (3D) assets in content creation to enhance visual effects and user interaction. Textures are critical in improving the visual realism of 3D geometric models. These 3D models must not only yield high-quality and diverse appearances but also enable fine-grained control over the generation process. Recent advances in generative artificial intelligence have introduced new paradigms for 3D content creation. In particular, denoising diffusion models [1, 2] have demonstrated remarkable performance in text-to-image generation tasks and have been effectively applied to texture-generation tasks.

Current text-to-image diffusion models [3] generate high-quality images that align well with textual descriptions. However, texture generation remains a significant challenge. It requires the generated texture to conform to the user’s prompts in appearance as well as fit the geometric surface and maintain consistency across multiple views. Hence, researchers have investigated various methods for generating high-quality textures, including score distillation sampling (SDS) [4] to indirectly leverage the pretrained diffusion model as a prior [5, 6], or directly generating multiview images with diffusion models and subsequently aggregating them into texture space [7, 8]. Nevertheless, most existing methods rely solely on text prompts as control conditions. Compared with text, images can convey user intent more intuitively and accurately. Existing image-guided texture-generation methods either achieve only style consistency while compromising appearance details from the reference image or require time-intensive fine-tuning to capture those details. Thus, achieving efficient and precisely controlled texture generation remains a significant challenge.

Therefore, for image-guided texture generation, we propose IP-ConTex, which is a novel texture-generation method that leverages a pretrained two-dimensional (2D) image diffusion model. IP-ConTex enables detail-preserving texture generation while maintaining multiview consistency without requiring fine-tuning or optimization. First, it uses a synchronized multiview diffusion framework [9] that shares the generated content across views in UV space at each denoising timestep, thereby ensuring structural and layout consistency across views. Subsequently, the appearance of the reference image is injected into the generation process via a novel appearance-control module. This module achieves fine-grained control and multiview consistency using four components: cross-attention control, self-attention control, diffusion feature synchronization, and color adjustment. Finally, experiments demonstrate that IP-ConTex can achieve stronger appearance preservation while maintaining competitive multiview consistency compared with existing state-of-the-art methods.

The main contributions of the proposed method are as follows:

- A novel image-guided texture generation method, IP-ConTex, is proposed, which introduces an appearance-control module to achieve fine-grained appearance control during texture generation. The method yields high-quality, detail-consistent textures for arbitrary 3D geometric models without fine-tuning or additional optimization.
- A diffusion feature-synchronization strategy is proposed, which fuses intermediate U-Net feature maps across views in UV space at each denoising timestep, thus enabling fine-grained appearance details to propagate consistently across all views and effectively suppressing blurring and ghosting artifacts in the final texture.
- A hybrid attention scheme combining cross-image self-attention and multiview shared attention is designed for the multiview texture-generation setting. It establishes semantic correspondence between the reference image and target views while promoting appearance consistency across views with considerable viewpoint disparities.

## Related studies

Transferring the appearance of a specified image into a 3D model is challenging. Early studies [10–12] on texture generation focused on synthesizing tileable texture maps from example patterns. Additionally, researchers [13, 14] investigated the correlation between geometric surface features and local texture variations, thus enabling geometry-aware texture synthesis. However, owing to inadequate perceptual understanding of both images and geometry, conventional methods fail to generate semantically rich textures on complex 3D structures. Meanwhile, owing to the rapid advancement of deep learning, convolutional neural networks have proven to be highly effective in image-related tasks. Various generative models, such as generative adversarial networks [15, 16], variational autoencoders [17, 18], and recent diffusion models[1, 2], have been developed and applied to texture-generation tasks. Some methods, including Texturify [19], Mesh2Tex [20], and PointUV [21], attempt to generate textures directly from 3D data by designing specialized texture representations for supervised learning. However, despite the abundance of 2D image data, high-quality 3D datasets are scarce and cannot be easily obtained and annotated. Therefore, the generalizability of these methods is limited owing to the low diversity of 3D training data. Recently, diffusion models trained on large-scale 2D image datasets have emerged as promising alternatives for high-quality 3D content generation. These models offer superior visual fidelity and flexible multimodal conditioning, thus rendering them suitable for image-guided texture generation.

### Using pretrained 2D diffusion models for texture generation

Owing to significant advancements in image generation, recent texture-generation methods have leveraged pretrained 2D diffusion models to overcome the scarcity of large-scale, high-quality 3D training datasets. These methods have significantly promoted the progress of texture generation, thereby enabling the generation of diverse and high-quality textures for 3D geometric models. Existing methods that utilize 2D diffusion models for texture generation can be broadly categorized into two groups.

The first category of methods uses SDS[4] or related techniques such as variational score distillation (VSD) [22] to indirectly employ the pretrained diffusion model as a prior. SDS enables the use of generative knowledge from diffusion models to optimize parameterized representations (e.g., neural radiance fields), thereby establishing the foundation for many recent 3D generation frameworks. These methods use views generated by diffusion models as supervisory signals to guide the optimization of texture parameters. One representative method is latent paint [5], which is among the first to apply SDS to latent diffusion models [3]. It propagates SDS-based gradients to a latent-space texture map through differentiable rendering. However, latent-space optimization at low resolutions typically results in blurring and loss of fine details in the generated RGB textures. Hence, researchers have proposed novel texture parameterizations to improve texture quality within the SDS-based optimization framework. For instance, Paint-it [23] introduces deep convolutional physically based rendering parameterization. Instead of directly optimizing pixel values in the texture map, it optimizes a neural proxy that can simulate the physical surface properties, thus yielding more realistic textures. SceneTex [24] proposes a multiresolution texture field to implicitly encode the appearance of 3D scenes, thereby enabling flexible learning of both low- and high-frequency appearance features. Despite these advancements, SDS-based optimization methods require long convergence times, yield overly saturated colors, and lack output diversity. Consequently, the resulting textures do not exhibit the quality and realism achieved through direct 2D image generation.

The second category of methods directly uses the diffusion model to denoise images rendered from multiple viewpoints and then back-projects the generated views into UV space to update the texture map. These methods typically incorporate geometric information—such as depth or normal maps—to guide or condition the denoising process, thereby ensuring alignment between the generated texture and the underlying 3D geometry. Specifically, the view images are rendered sequentially from different camera positions. At each viewpoint, a 2D diffusion model performs inpainting based on partial content from neighboring views, thus allowing more detailed textures to be generated while improving computational efficiency. For example, TEXTure [7] and Text2Tex [8] progressively generate local textures from multiple predefined viewpoints. Missing regions are inpainted, and the inpainted results are then backprojected into the texture space to iteratively refine the texture map. However, because of the asynchronous denoising of multiple views, the resulting texture lacks global consistency constraints. Consequently, this progressive texture update strategy results in inconsistencies across views and thus visible seams. Moreover, these methods are susceptible to the Janus problem, wherein similar frontal appearances are generated for both the front and back views, thereby resulting in unrealistic ‘multi-faced’ textures. Additionally, the generated views may contain inconsistent lighting, thus hindering their applicability in downstream applications such as relighting or physically based rendering. Hence, researchers have proposed improved approaches within this category of methods. Paint3D [25] trains a diffusion model directly in UV space and adopts a coarse-to-fine texture-generation strategy. Initially, images generated from each viewpoint are used as the initial texture map. Subsequently, a position map of the geometric model is used as a conditioning input to refine the texture map and remove lighting information. Similarly, Meta 3D TextureGen [26] focuses on refining texture maps in UV space. MVPaint [27] introduces a multiview generation model that first generates synchronized multiview images to mitigate the Janus problem. Subsequently, it fills in unobserved regions in the 3D point cloud, thereby minimizing dependence on the continuity of UV unwrapping. Finally, it applies a super-resolution model and seam-smoothing algorithm to further enhance texture quality. Unlike methods such as TEXTure, some recent methods conduct multiview denoising and texture aggregation synchronously to promote multiview consistency. For example, TexFusion [28] iteratively denoises each view at every timestep and updates a shared latent texture map accordingly. Meanwhile, SyncMVD [9] fuses denoised content from all views at each denoising timestep. These methods synchronize multiview denoising on a shared texture map, thus effectively improving the multiview consistency of the generated texture.

### Generating textures with image prompts

Most existing methods focus on generating textures guided by textual prompts. However, accurately describing complex and fine-grained patterns using only text is challenging. By contrast, images provide a more intuitive method for conveying user intent. Several methods have been proposed to investigate image-guided texture generation. TEXTure [7] proposes a method to extract texture information from an existing textured model or several images. It employs text inversion [29] to learn a specialized token that represents the reference appearance. This token, along with the reference images, is then used to fine-tune the pretrained diffusion model using DreamBooth [30], thereby enabling the generation of personalized textures for new 3D models. Similarly, TextureDreamer [31] applies DreamBooth fine-tuning to extract appearance features from sparse input images and then uses VSD optimization to transfer textures onto arbitrary 3D geometries. In addition to fine-tuning-based methods, other methods such as Paint3D [25] and FlexiTex [32] utilize IP-Adapter [33] to inject appearance information from reference images directly into the diffusion model during the generation process. StyleTex [34] extracts style features from a reference image to guide consistent texture generation. AlignTex [35] trains an AlignNet to align generated images with the input multiview artwork and a 3D mesh. TexTrans [36] utilizes multiview reference images to transfer 3D textures.

Existing image-guided texture-generation methods can effectively extract appearance information from reference images. However, these methods either incur additional computational costs owing to fine-tuning or only achieve style-level consistency, thus rendering it difficult to preserve fine-grained appearance details. In 2D image generation and editing, some methods generate images that are consistent with the appearance of a reference image [37–39] or perform complex nonrigid editing [40–42]. Although these approaches successfully preserve appearance details, they are typically limited to single-image applications and cannot be generalized to 3D domains. By contrast, 3D texture generation requires not only conformity to the reference image but also 3D consistency across multiple views. Hence, we propose a novel method that enables image-guided detail-consistent texture generation without requiring fine-tuning or additional optimization, thereby improving both efficiency and consistency. Unlike prior approaches that either apply appearance control to single images or adopt image prompts only through cross-attention, our method introduces a dedicated feature-level synchronization mechanism and a hybrid attention design that accommodate the geometric constraints of multiview texture generation.

## Methods

### Problem setting and overview

#### Problem statement

The goal of this study is to synthesize a high-quality texture map $\mathcal{T}$ for a 3D geometric model $\mathcal{M}$, guided by a reference image $\mathcal{I}_{ref}$ and textual prompt $\mathcal{P}$. The generated texture $\mathcal{T}$ must not only align with the geometric surface of $\mathcal{M}$ but also consistently preserve the fine-grained appearance details and stylistic features of $\mathcal{I}_{ref}$ across multiple views.

#### Definition of texture

Herein, we use the term *texture* to refer to a UV-mapped appearance field defined on the surface of a 3D geometric model. It specifies the surface color pattern used to render objects from different perspectives. This notion is distinct from the model geometry, physically based reflectance properties—such as BRDF parameters—and the rendered 2D image. In our method, the final output is a texture map in UV space, whereas the rendered multiview images are intermediate observations used to synthesize and aggregate the surface appearance.

To achieve this, we utilized a pretrained 2D image diffusion model to perform multiview generation without domain-specific training. First, a set of *N* predetermined viewpoints $\{v_1, v_2, \dots, v_N\}$ is positioned around the model to capture its surface. Depth maps rendered from these viewpoints can serve as control conditions for a 2D image diffusion model to generate view-specific images. To initialize texture generation, a latent texture map $W_t$ is sampled from a standard normal distribution. Subsequently, this noise map is projected onto each viewpoint to obtain the initial latent representation $Z_T=\left \{z_{T}^{(v_i)}\right \}^{N}_{i=1}$ corresponding to these views. Guided by the text prompt and depth map of each viewpoint, view images $\left \{z_{t}^{(v_i)}\right \}$ are generated using the text-to-image diffusion model. The generated views are back-projected onto the 3D surface and mapped onto UV space to obtain a texture map.

**Fig. 1:**
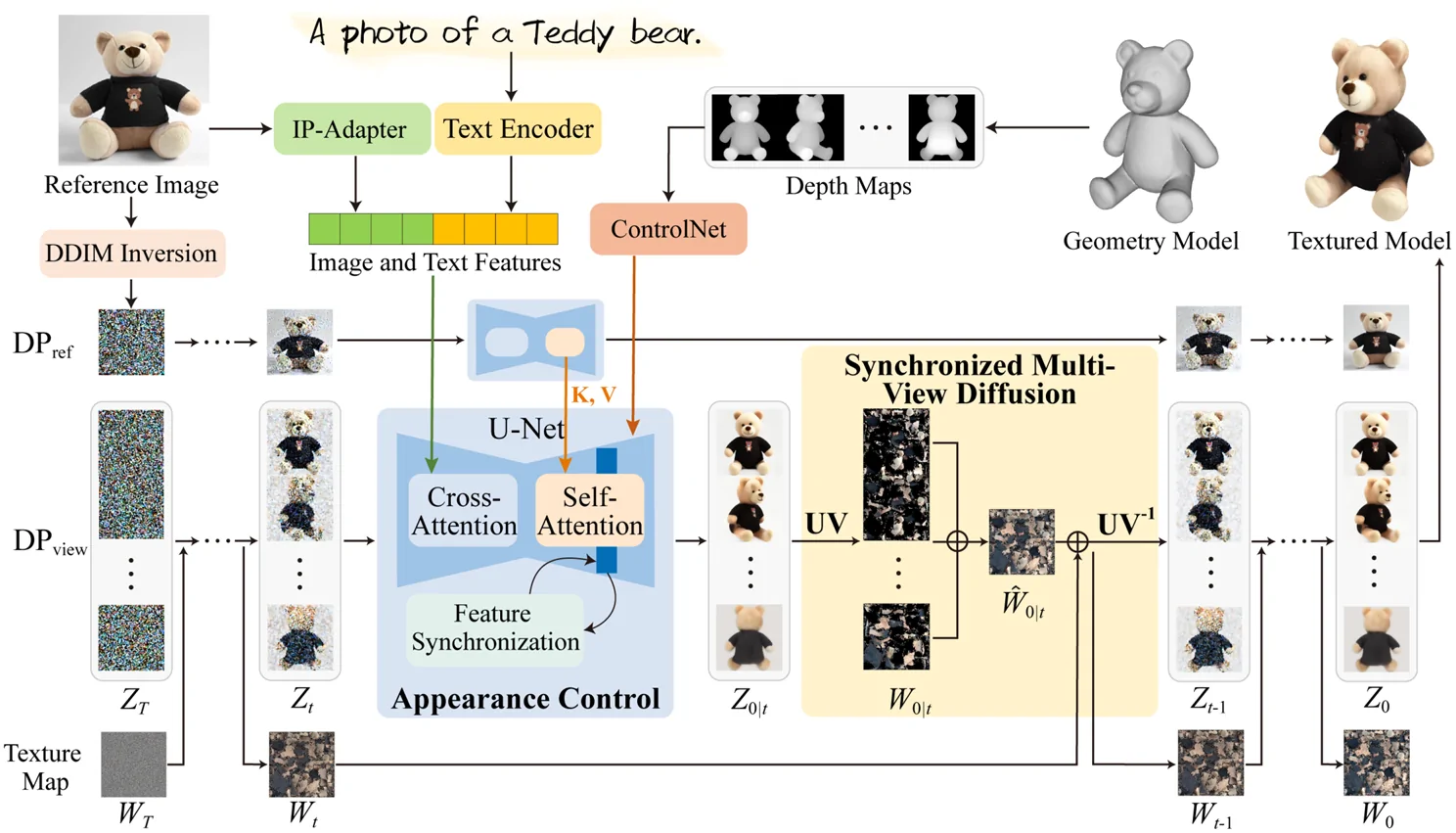
Framework of IP-ConTex. For a reference image, geometric model, and simple text prompt, IP-ConTex generates high-quality textures that are detail consistent with the reference image. The framework has two key modules: a synchronized multiview diffusion module, which ensures content consistency acrossa small constant for avoiding division by zero multiple viewpoints; and an appearance-control module, which injects appearance features from the reference image into the generation process

The main challenge in image-guided texture generation is the generation of visually consistent images across views while preserving fine details. Hence, we propose IP-ConTex, a detail-consistent texture-generation method that does not require fine-tuning or additional optimization. It comprises two key modules: a synchronized multiview diffusion module and an appearance-control module. The synchronized multiview diffusion introduced by SyncMVD [9] shares denoising information across views at each timestep to ensure structural and layout consistency. In this study, we focus on generating textures that are consistent with the appearance details in a reference image. Thus, we design an appearance-control module that injects the visual features of the reference image into the texture-generation process to preserve the appearance details. The overall framework of IP-ConTex is shown in Fig. 1. The details of the synchronized multiview diffusion and appearance-control modules are presented in subsections “Synchronized multiview diffusion” and “Appearance control”, respectively.

#### Terminology on consistency

To avoid ambiguity, we distinguish three related but different notions herein. Style consistency refers to the similarity of global visual characteristics—such as overall color tone or artistic style—between the generated result and reference image. Appearance preservation refers to the consistent transfer of specific visual attributes from the reference image, including local patterns, fine-grained details, and characteristic surface structures. 3D consistency refers to the agreement of generated content across viewpoints, such that the same surface region of the 3D model is assigned compatible appearance when observed from different views.

### Synchronized multiview diffusion

If each view is generated independently, then the resulting content may become inconsistent, thus resulting in prominent artifacts in the final texture. To mitigate multiview inconsistency, our method adopts the synchronized multiview diffusion framework introduced in SyncMVD[9]. Accordingly, we employ a synchronized multiview diffusion module to share latent information across all views at each denoising step.

As shown in Fig. 1, at timestep *t*, the diffusion model computes the noise-free images $Z_{0|t}$ for each view, along with the input latent representation $Z_{t-1}$ for the next timestep *t-1*. To ensure consistency among the latent images $Z_{t-1}$, the noise-free images $Z_{0|t}$ are first projected onto the UV space and then fused to obtain a latent representation $W_{0|t}$, which represents the noise-free texture map at timestep *t*. Subsequently, $W_{0|t}$ is combined with the noisy texture map $W_t$ input at the current timestep to compute the denoised texture map $W_{t-1}$. This denoised texture map is then re-projected onto each view to generate consistent latent views $Z_{t-1} = \mathrm{UV^{-1}}(W_{t-1})$, which are used as inputs for the next denoising timestep *t-1*. The synchronized noise-free texture map $\hat{W}_{0|t}$ at timestep *t* is computed as follows:

1 $$\hat{W}_{0|t} = \frac{\sum_{i=1}^{N} \theta^{(v_i)} \odot UV(z^{(v_i)}_{0|t})}{\sum_{i=1}^{N} \theta^{(v_i)} + \gamma} $$

where *⊙* denotes the element-wise multiplication of the pixel values. The operator $\mathrm{UV}(\cdot)$ maps image-space latents to the UV domain using bilinear interpolation based on the rasterized mesh surface. $\theta ^{(v_i) }$ is the weight assigned to each pixel from viewpoint $v_i$, which is calculated based on the cosine of the angle between the face normal and viewing direction. The exponent *α* is used to adjust the smoothness of the fused texture map. Specifically, *α* adjusts the tradeoff between multiview consistency and high-frequency details. A smaller *α* results in a greater average contribution from each view, which facilitates consistency across views. By contrast, a larger *α* tends to preserve the high-frequency details of each view. Therefore, a smaller *α* can be used in the early stages to ensure multiview consistency, whereas a gradually increasing *α* helps preserve fine-grained texture details as denoising progresses. Additionally, *γ* is a small constant for avoiding division by zero.

To ensure that only valid surface regions are updated from each viewpoint, we compute a visibility mask $M^{(v_i)}$ for each camera. This is implemented by verifying the pixel-to-face mapping from the rasterizer to identify triangles that are neither occluded nor back-facing. The fusion weight $\theta^{(v_i)}$ in Eq. 1 can be expressed as

2 $$\theta^{(v_i)} = M^{(v_i)} \cdot \max(0, \cos(\mathbf{n}, \mathbf{d}_i))^\alpha $$

where $\mathbf{n}$ is the face normal and $\mathbf{d}_i$ is the viewing direction. This weighting strategy prioritizes views in which the surface is more orthogonal to the camera, thus significantly reducing stretching artifacts at grazing angles.

Synchronized multiview diffusion fuses the overlapping regions among different views by sharing content in the UV space at each denoising timestep. This ensures that the overall structure and color distribution across views remain consistent. Specifically, we perform synchronized multiview denoising only during timestep $t > \tau_{sync}\text {,}$ which is sufficient to ensure structural and stylistic consistency across views. In the subsequent timesteps, denoising is performed in the screen space, where fine-grained appearance control is performed to transfer the appearance from the reference image.

### Appearance control

The goal of appearance control is to generate textures that exhibit the appearance of an object in the input reference image while preserving fine-grained visual details. Recent studies [39, 43, 44] demonstrated that the cross- and self-attention mechanisms within a denoising network encode rich semantic information regarding the generated image. By leveraging the semantic understanding capabilities of the diffusion network, the proposed appearance-control module injects appearance information from the reference image into the texture-generation process.

**Fig. 2:**
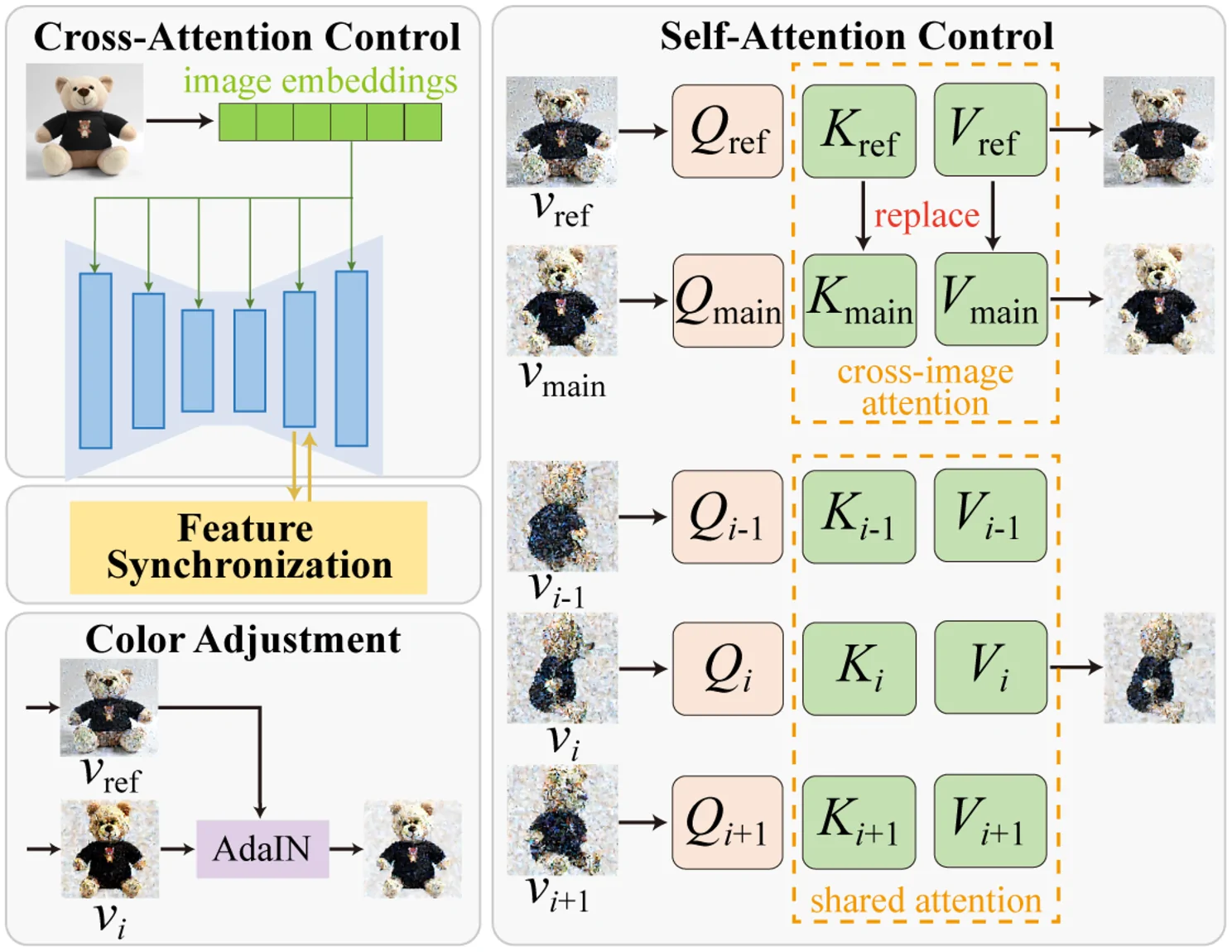
Four components of the appearance-control module

The appearance-control module comprises four components, as shown in Fig. 2. First, we apply cross-attention control using IP-Adapter [33] to establish the initial structural layout and style of the texture during the early denoising stages. The generated content is synchronized across different views using the synchronized multiview diffusion framework introduced in subsection “Synchronized multiview diffusion”. Second, self-attention control retrieves content from the reference image and then injects it into the target view to preserve the appearance details. Third, to ensure the consistent propagation of appearance details across views, we propose a diffusion feature-synchronization method. Finally, we perform color adjustment using adaptive instance normalization (AdaIN) [45] to align the color distribution of the generated latent-view images with that of the reference image at each timestep. The following sections provide detailed explanations of these four components.

#### Cross-attention control

Recent studies [43] demonstrated that cross-attention maps typically encode conceptual information regarding an object and are associated with the semantics of input prompts, whereas self-attention maps capture shape, structure, and fine-grained visual details. IP-Adapter [33] is a lightweight image prompt adapter designed for Stable Diffusion models. It employs a decoupled cross-attention mechanism that allows images to be used directly as prompts to control both the style and semantics of the generated images. An additional cross-attention block is integrated to process the image features in each cross-attention layer of the U-Net diffusion model.

Within the synchronized multiview diffusion framework, the structural layout of each target view is approximately established during the early denoising stages. Although a synchronized diffusion strategy enables information exchange across overlapping regions between adjacent views, eliminating inconsistencies in the structural layout or color style across different views remains challenging. Since the structure and style established in the early denoising stage significantly affect the final image appearance, our method injects appearance information from the reference image into the generation process via IP-Adapter and applies it to all views. Compared with text prompts, image prompts provide more explicit and detailed guidance. Therefore, the denoising of each view is guided more consistently, thus promoting global consistency of semantics and style across views. However, using IP-Adapter may introduce new visual features that can compromise appearance consistency. Therefore, IP-ConTex restricts cross-attention control to timesteps $t > \tau_{cross}$.

#### Self-attention control

Although IP-Adapter can generate images with stylistic similarity to the reference image, it fails to synthesize highly consistent appearance details. Previous studies [39, 40] demonstrated that modifying the key and value features of the self-attention layer offers finer control over the generated content and enables the transfer of detailed appearance information between images. Therefore, we introduce cross-image attention based on the self-attention layer of the diffusion model to implicitly establish semantic correspondences between images. Specifically, we use the Query features from the target view image, and the key and value features from the reference image. This cross-image attention allows the diffusion model to establish semantic correspondence between two images, thereby injecting detailed appearance information from the reference image into the target view during the generation process.

First, the input reference image is reversed into a noisy image using DDIM inversion [46] to obtain its initial latent representation $z_{ref}$. This serves as the input at timestep *T* for the denoising process $\mathrm{DP}_{\mathrm{ref}}$, which reconstructs the reference image. Next, $\mathrm{DP}_{\mathrm{ref}}$ is used to guide the generation of the main view (assumed to be the front view of the object, as shown in Fig. 1). During the denoising process $\mathrm{DP} _{\mathrm{view} }^{(v_1)}$, which generates the target main view, the query features ($Q_{\mathrm{main}}$) from its self-attention layer are used to query the key ($K_{\mathrm{ref}}$) and value ($V_{\mathrm{ref}}$) features from the corresponding self-attention layer in $\mathrm{DP}_{\mathrm{ref}}$. This cross-image attention strategy utilizes the content of the reference image as the material, thereby preserving user-specified appearance details. Simultaneously, the structural layout of the generated image is governed by the depth map of the main view, thus ensuring that the generated texture aligns with the semantics of the geometric surface.

For target views without corresponding reference images (i.e., non-main views), the substantial disparity renders it ineffective to directly replace *K* and *V* of the self-attention layer with those of the reference image. Prior studies [47, 48] demonstrated that arranging multiple views of an object within a single image during denoising results in a more consistent appearance generation. To further enhance appearance consistency across views, each non-main target view attends not only to itself but also to its adjacent views. Accordingly, IP-ConTex employs a shared attention strategy and view concatenation within the self-attention module to promote consistency in the multiview appearance.

Therefore, the self-attention of the different target views is calculated as follows:

3 $$\mathrm{Attention}_{\mathrm{self} }(v_i, l)= \begin{cases} \mathrm{SA}(Q_i, \{K_{ref}, V_{ref}\}), & \text{if } l \in L_{self} \text{and } \\ & v_i\text{is a main view } \\ \mathrm{SA}(Q_i, \{K_j, V_j\}_{j \in \mathcal{N}_i}),& \text{otherwise } \end{cases}$$

where *l* denotes the layer index of the U-Net. The function $\mathrm{SA}(Q, \{K, V\})$ represents the self-attention operation. For non-main views, $\{K_j, V_j\}_{j \in \mathcal{N}_i}$ denotes that the Key and Value features are concatenated from the set of adjacent views $\mathcal{N}_i = \{v_{i-1}, v_i, v_{i+1}\}$ along the spatial dimension. The input reference image is denoted by $v_{\mathrm{ref}}$. The main view refers to the view with the corresponding reference image. $v_{i-1}$ and $v_{i+1}$ are the left and right adjacent views of $v_{i}$, respectively. Therefore, the first line of Eq. 3 corresponds to cross-image attention, where content is queried from the reference image. The second case corresponds to shared attention, which promotes appearance consistency across multiple views. Notably, cross-image attention is applied only at the $L_{self}$ layer of the U-Net for the target main view. This restriction is based on the hierarchical feature distribution within the diffusion model’s U-Net architecture. The encoder and bottleneck layers primarily encode high-level semantic layouts and global structural information. Injecting reference features at these early stages forces the target view to inherit the global geometry of the reference image, thus conflicting with the spatial constraints imposed by the target 3D model’s depth map. By contrast, the decoder layers—specifically the later stages—synthesize localized visual details and high-frequency textures. Consequently, applying this cross-image attention to all the layers causes the generated image to closely resemble the reference image, thereby failing to align with the semantics of the geometric surface. Prior studies [40] demonstrated that performing appearance injection from the reference image through the self-attention layer in the U-Net decoder enables better transfer of the desired content while preserving the structural layout of the target view.

Through the two self-attention mechanisms described above, namely cross-image self-attention and shared self-attention, the appearance details of the reference image can be effectively injected into the generation process of the target main view and propagated to other target views.

#### Diffusion feature synchronization

By controlling the cross-attention and self-attention during the denoising of the target views, the appearance information from the reference image can be effectively injected into the generated views. Moreover, attending to adjacent views further enhances the appearance consistency across all views. In texture generation, 3D consistency must be maintained across views, which implies that the content corresponding to the same spatial location of the 3D model should remain consistent across different views. Otherwise, blurring and ghosting artifacts will emerge in the final aggregated texture.

The synchronized multiview diffusion strategy described in subsection “Synchronized multiview diffusion” is applied only during the early denoising stages to rapidly establish structural consistency across views. Because the appearance details are gradually refined throughout the denoising process, maintaining 3D consistency during denoising is essential. Hence, we propose a diffusion feature-synchronization strategy to promote 3D consistency of appearance details across multiple views.

The intermediate features of the U-Net within the diffusion model contain rich semantic and structural information regarding the images. Prior studies [41] demonstrated that different layers of the U-Net decoder capture different levels of detail; the lower layers primarily capture coarse semantic information, whereas the higher layers capture finer appearance details. Based on these insights, we apply a synchronization strategy to the diffusion features of each view. Specifically, when the timestep $t > \tau_{feat}$ and the number of U-Net layers is $l\in L_{feat}$, we extract the intermediate feature maps of each view from the diffusion network. These features are fused using the method expressed in Eq. 1, reprojected to each view, and then used to replace the original intermediate feature maps within the network. This process can promote the 3D consistency of appearance details across multiple views.

#### Color adjustment

After applying the aforementioned appearance control, we can obtain latent images corresponding to both one-step and fully denoised outputs—$\left \{z_t^{(v_i)} \right \}$ and $\left \{z_{0|t}^{(v_i)}\right \}$, respectively—at each timestep. However, we observed a discrepancy in the color distribution between the generated and reference images. Hence, we adopted AdaIN [45] to adjust the color distribution of the output latent images. AdaIN was originally developed for style-transfer tasks and can effectively align the feature statistics between latent representations. Therefore, at each timestep *t*, each view $z_t$ is updated using the transformation $z_t^{(v_i)}\gets \mathrm{AdaIN} (z_t^{(v_i)}, z_{\mathrm{ref}})$ to align the feature statistics of $z_t$ with those of the reference latent image $z_{\mathrm{ref}}$. This operation effectively harmonizes the color distribution across the views.

## Results

This section presents the experimental results and an analysis of the proposed texture-generation method, IP-ConTex. We first introduce the key implementation details, including the parameter settings and experimental configurations. Next, we compare IP-ConTex with three state-of-the-art image-guided texture-generation methods. The effectiveness of the core modules is evaluated via ablation studies. Finally, we analyze the time efficiency of the proposed method.

### Implementation details

IP-ConTex is developed based on the pretrained diffusion model Stable Diffusion v1.5 and integrates ControlNet [49] and IP-Adapter [33] to receive depth-map conditions and image prompts, respectively. The denoising process follows the DDIM [46] scheduler. For a specified geometric model, 11 predefined views are used for multiview diffusion generation. Among them, eight views are evenly distributed around the object at an elevation angle of $0^{\circ}$, with the view corresponding to the reference image denoted as the main view. Additionally, two top views at an elevation angle of $30^{\circ}$ are positioned in the direction of the main view and its opposite, respectively, along with a single bottom view.

We implemented projection and back-projection operations between the view images and UV texture map using PyTorch3D [50]. For meshes without predefined UV coordinates, we first unwrapped the input mesh using xatlas to obtain the UV vertices and UV face indices. For each predefined viewpoint, an orthographic camera was constructed from the corresponding elevation and azimuthal angles. We rasterized the mesh from each camera view and used the pixel-to-face buffer to determine the visible triangles. Subsequently, the visibility information was transferred to the UV domain to obtain a per-view UV visibility mask. During texture baking, each generated view was projected onto the UV texture space based on the rasterized face indices and barycentric coordinates. To fuse multiple views, we computed a cosine weight for each visible texel based on the angle between the surface normal and viewing direction. Views that observed a surface more frontally were assigned greater weights, whereas grazing-angle observations contributed less.

In all experiments, the number of sampling steps *T* was set to 50, and the classifier-free guidance coefficient was fixed at 7.5. For the synchronized multiview diffusion module, $\tau_{sync}$ was set to 0.6 to promote the consistency of the structural layout and style during the early denoising stages. The texture-map fusion smoothness parameter $\alpha_{tex}$ was increased linearly from 1.0 to 6.0, thus allowing the early denoising stages to emphasize cross-view consistency while preserving high-frequency details in the later stages.

For cross-attention control in the appearance-control module, $\tau_{cross}$ was set to 0.2, thus enabling the image appearance extracted by IP-Adapter to be injected into each view’s generation process at the beginning of denoising. Self-attention control was applied throughout the denoising process to preserve the appearance details. The U-Net architecture of Stable Diffusion comprised 16 attention layers (indexed from 0 to 15), which included the encoder, bottleneck, and decoder. Prior studies [40] demonstrated that applying self-attention control across all layers results in generated images that closely resemble the source image, thereby failing to fit the structural layout of the target. Effective appearance transfer can be achieved when the control is restricted to the decoder layers. Accordingly, following the configuration in MasaCtrl [40], $L_{self}$ was set to layers 10 through 15.

For the diffusion feature synchronization, $\tau_{feat}$ was set to 0.8, and the fusion smoothness parameter $\alpha_{feat}$ was set to 6.0. We observed that by *t = 0.8*, the structural layout and fine details of each view were primarily established. Consequently, feature synchronization was unnecessary during the final timesteps. We used the original diffusion network to further refine image quality and realism. The U-Net decoder comprises four layers at different scales indexed from zero to three, each of which focuses on extracting features at different levels. Layer 2 emphasizes low-level details [51]. Therefore, in our method, $L_{feat}$ was set to *2* to synchronize fine-grained features across views.

IP-ConTex is designed for texture generation guided by image prompts. To evaluate its effectiveness, we compared it with three state-of-the-art methods: TEXTure [7], Paint3D [25], and PGC-3D [6] to include the primary technical routes of image-guided texture generation. TEXTure sequentially generates multiview images to progressively update the texture. It utilizes DreamBooth [30] to fine-tune the pretrained diffusion model and extract appearance information from the reference image. Similarly, Paint3D adopts a progressive multiview generation strategy but further refines the initial texture map using a diffusion model trained in UV space. Additionally, Paint3D uses IP-Adapter for image-guided generation. PGC-3D is an optimization-based method that uses SDS to optimize texture parameters. The geometric models used in the experiments are sourced from the Objaverse dataset [52]. For a quantitative evaluation, we constructed 50 test samples, where each sample comprised a mesh model and a corresponding reference image. The mesh models were selected from the Objaverse dataset and spanned various object categories, including animals, clothing, and toys. For each mesh model, several reference images were retrieved from the Internet and used separately to guide texture generation. Collectively, these baselines include fine-tuning-based image guidance, feedforward image guidance, and optimization-based texture generation. All experiments were conducted on a single NVIDIA GeForce RTX 3090 GPU.

### Texture results

**Fig. 3:**
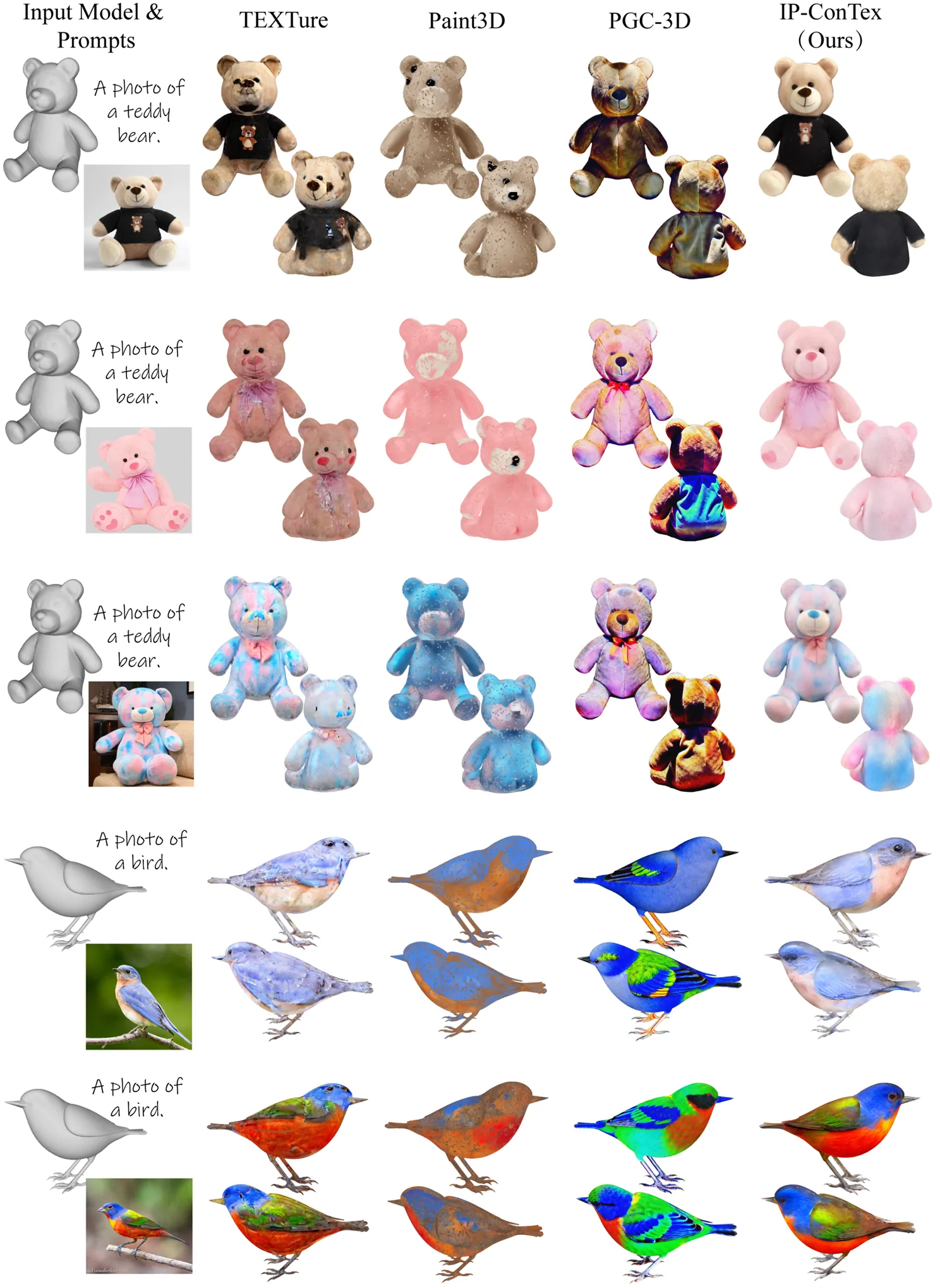
Image-guided texture-generation results and comparison (1). Results are shown for three bear models and two bird models. The first column displays the input geometric model and reference image. The subsequent columns present the texture-generation results from TEXTure [7], Paint3D [25], PGC-3D [6], and the proposed IP-ConTex. Each model is visualized from two different viewpoints

**Fig. 4:**
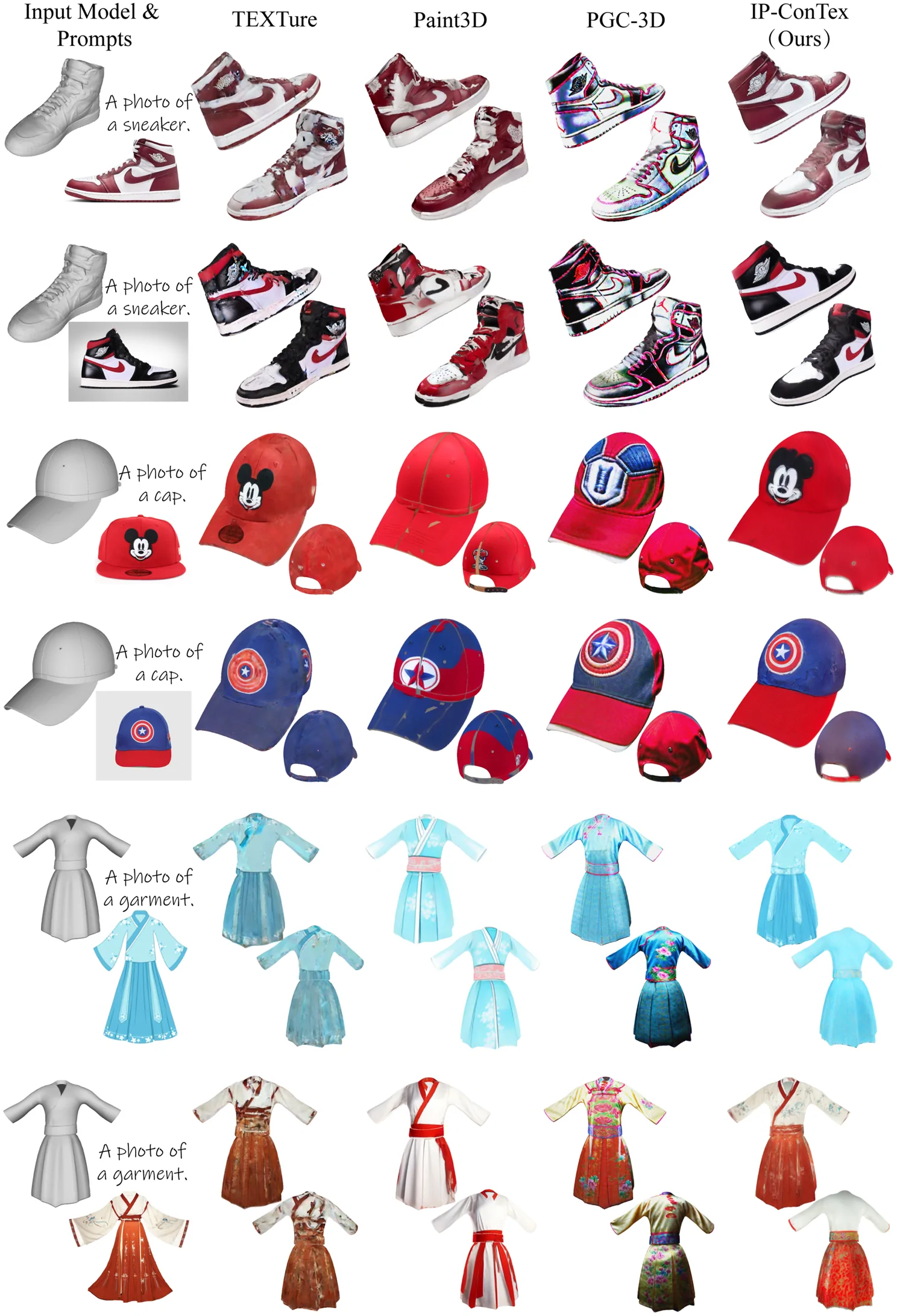
Image-guided texture-generation results and comparison (2). Results are shown for shoe, cap, and clothing models. The first column displays the input geometric model and reference image. The subsequent columns present the texture-generation results from TEXTure [7], Paint3D [25], PGC-3D [6], and the proposed IP-ConTex. Each model is visualized from two different viewpoints

Based on using the geometric model and reference image as inputs, Figs. 3 and 4 present the texture-generation results yielded by IP-ConTex, along with those yielded by three state-of-the-art methods: TEXTure [7], Paint3D [25], and PGC-3D [6].

TEXTure generates views sequentially through progressive inpainting, thus resulting in fragmented textures and prominent seam artifacts, as shown in the shoe model in Fig. 4. Consequently, clear and refined textures could not be easily obtained. Additionally, TEXTure is susceptible to the Janus problem, as shown in the bear model in Fig. 3, where front-facing appearance appears on both the front and back of the object. Although TEXTure effectively captures fine-grained features from the reference image, its overall texture quality remains unsatisfactory. Furthermore, TEXTure requires fine-tuning of the diffusion model for each sample, which is time-consuming. Paint3D adopts progressive generation to obtain an initial texture map and is similarly susceptible to the Janus problem. It subsequently refines the texture using a diffusion model trained in UV space. However, its performance degenerates when complex UV mappings are involved, particularly when the UV space contains many discontinuities, as shown in the bear and bird models in Fig. 3. By contrast, models with simpler UV layouts, such as the clothing model shown in Fig. 4, yield better results. Although Paint3D and our method utilize UV space, both methods rely on it in fundamentally different manners. Paint3D performs texture refinement directly in the UV space and is thus sensitive to the continuity and regularity of UV parameterization. By contrast, our method synthesizes appearance in a rendered multiview image space and uses the UV space primarily for synchronization and aggregation across views. Consequently, severe UV seams or fragmented UV charts exert less effect on the generation quality of the proposed method. This robustness to topological complexity and surface discontinuities aligns with broader research challenges commonly addressed through subdivision surfaces and geometry-simulation decoupling [53–57]. Paint3D uses IP-Adapter to inject the reference-image appearance into the generation process via cross-attention, thus ensuring style and semantic consistency. Nevertheless, it fails to preserve fine appearance details. Finally, owing to the limitations of SDS, textures generated by PGC-3D tend to exhibit overly saturated colors and low consistency with the reference image, i.e., colors and patterns that differ significantly from the input are typically generated.

The proposed IP-ConTex adopts a synchronized multiview diffusion [9] framework to promote structural and layout consistency across different views, thus resulting in significantly improved texture quality. During generation, we followed SyncMVD [9] by adding directional text prompts to each view, which effectively alleviated the Janus problem. For image guidance, IP-ConTex integrates an appearance-control module that does not require fine-tuning. It relies on simple attention control and a feature-synchronization strategy to effectively extract appearance details from the reference image and then transfers them to a geometric model. Thus, IP-ConTex not only generates textures that highly resemble the reference image but also achieves more natural colors and richer details, thereby offering notable advantages in terms of appearance fidelity and 3D consistency.

**Fig. 5:**
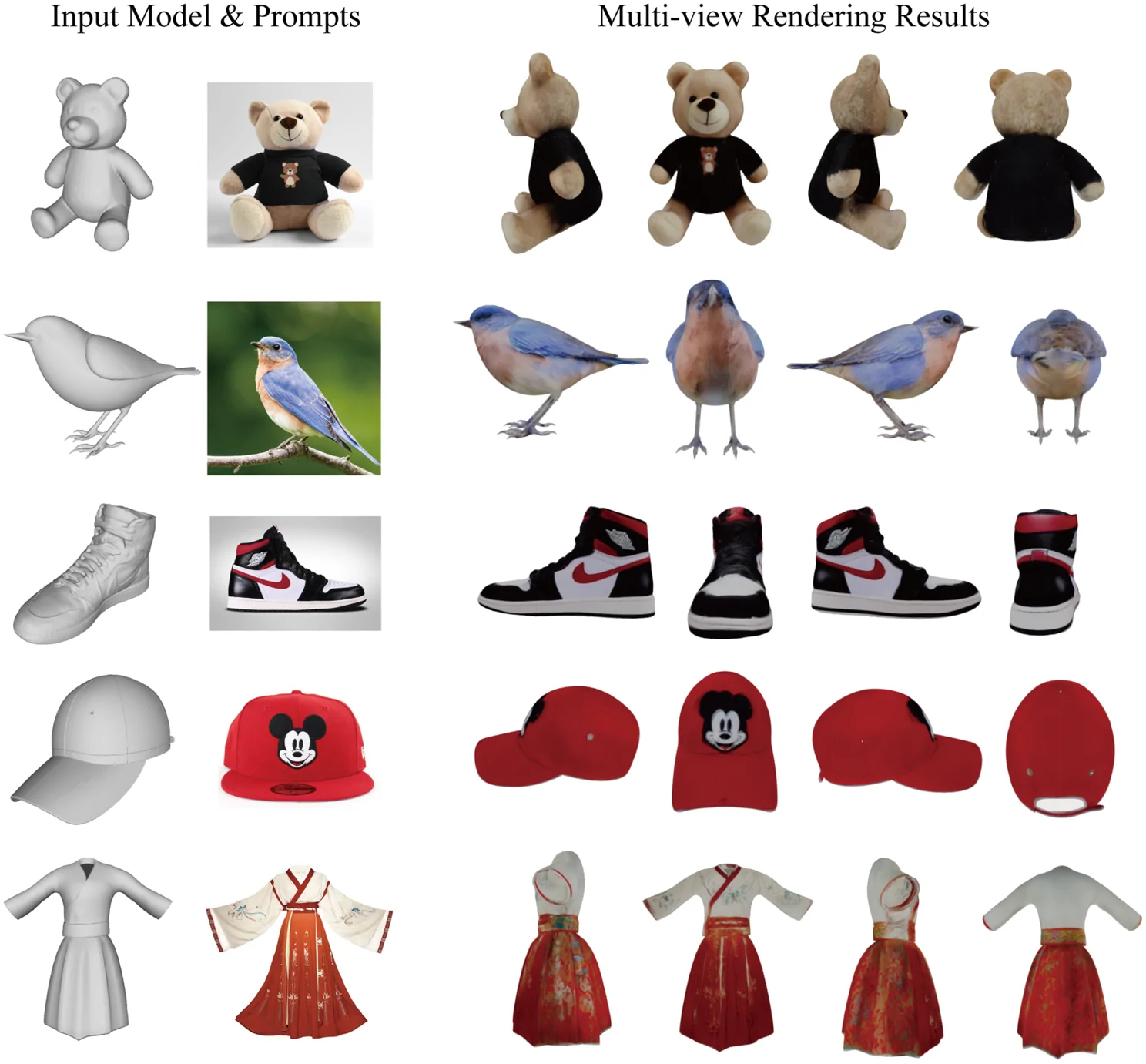
Multiview consistency and backside generation results. Texture-generation results are shown for five representative models, each rendered from four orthogonal viewpoints (front, back, left, and right). These results demonstrate that IP-ConTex maintains high appearance fidelity and 3D consistency across all directions. Even in unobserved regions (e.g., the backside), our method yields natural textures

To further evaluate the 3D consistency and the quality of texture generation in unobserved regions (e.g., the backside), we present expanded visual results for five representative models in Fig. 5. Each model is rendered from four orthogonal viewpoints: front, back, left, and right. These results demonstrate that IP-ConTex maintains high structural coherence and detailed consistency across all views. Specifically, the synchronized multiview diffusion effectively prevents the Janus problem, thus ensuring that the back of the models (such as the bear and bird) reflects the expected biological or structural layout instead of duplicating front-facing features. Moreover, the transition between views is smooth, with no significant seam artifacts or ghosting, thereby validating the effectiveness of our diffusion feature-synchronization strategy.

### Ablation study

To evaluate the effectiveness of the proposed appearance-control module for texture generation, this section presents a series of ablation experiments. By comparing the texture-generation results with and without each control component, we analyze the individual contribution of each component relative to the overall generation performance.

#### Cross-attention control

**Fig. 6:**
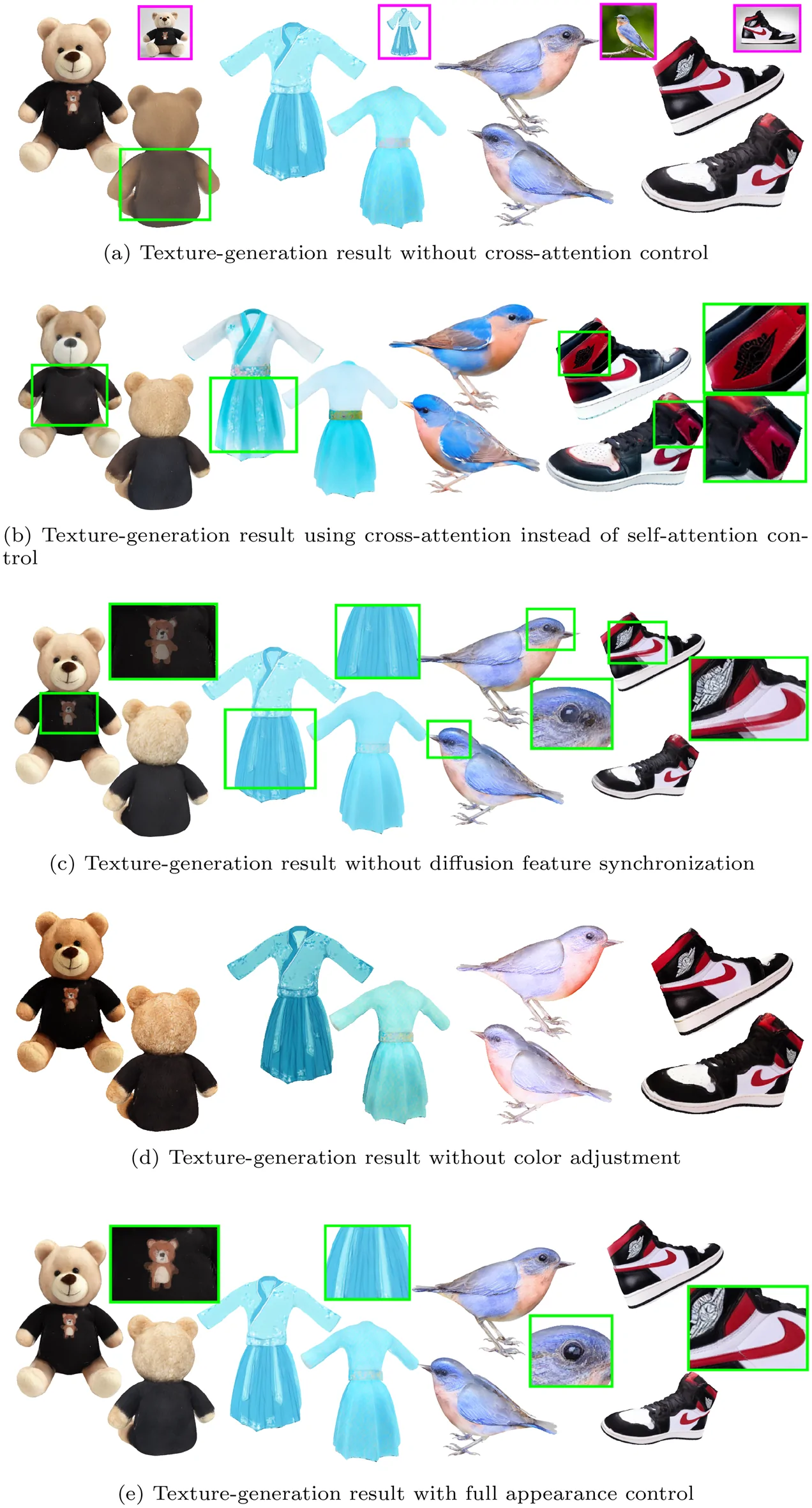
Results of ablation experiments on appearance-control module. For each model, the magenta rectangle shows input reference image. Each model is visualized from two viewpoints

IP-ConTex utilizes IP-Adapter to embed reference-image information into the cross-attention layer, thus enabling appearance features from the reference image to be injected into the generation process during the early denoising stages. Texture results without cross-attention control are shown in Fig. 6a. Notably, the back of the bear model failed to reproduce the expected clothing color and structure. By contrast, the other three models exhibited minimal changes. This is because the bird and shoe models exhibit mirrored appearances on both the front and back, and reference images are available for these views during generation. Additionally, the back of the clothing model has a relatively simple structure. Therefore, when the structure varies significantly between the views and reference images, cross-attention control is particularly important. This enables the extraction of semantic information from the reference image and affects both the structural layout and color styling during the early denoising stage.

#### Self-attention control

In addition to injecting reference-image information through cross-attention control, IP-ConTex employs self-attention control to query the content from the reference image during the generation of each view. Figure 6b shows the texture-generation results when cross-attention control is used instead of self-attention control. Under this setting, both the bear and clothing models failed to reproduce the key appearance details in the reference image, whereas the bird and shoe models generated texture patterns that were inconsistent with the reference image. These results demonstrate that cross-attention is insufficient for transferring fine-grained image features. By contrast, self-attention control is crucial for preserving the detailed appearance characteristics and ensuring fidelity to the reference image.

#### Diffusion feature synchronization

IP-ConTex uses diffusion feature synchronization during the generation of each view to align appearance details and ensure 3D consistency across views. Figure 6c shows the texture-generation results without feature synchronization. The bear and clothing models exhibited blurred textures and loss of fine details. This degradation occurred because detailed features from the reference image are generated only in the main view and fail to propagate to other views, thus resulting in an averaging effect of high-frequency details during the final texture-map aggregation. Moreover, the bird and shoe models exhibited ghosting artifacts, which is attributable to inadequate 3D consistency. The content generated in each view is inconsistently mapped to the same surface regions of the model. These results demonstrate that the proposed diffusion feature-synchronization strategy is essential for effectively propagating high-frequency appearance details across views and maintaining 3D-consistent textures.

#### Color adjustment

After each denoising timestep, IP-ConTex applies AdaIN to align the feature statistics of each view’s latent representation with those of the reference image. This adjustment ensures that the latent images better match the color distribution of the reference. As shown in Fig. 6d, omitting AdaIN results in textures with color tones that deviate from the reference image. By contrast, applying AdaIN results in textures with color distributions that are more consistent with the reference. This may be because the latent images of each view are aggregated in the UV space and then projected onto each view. This process may change the feature statistics. Therefore, AdaIN is essential for maintaining color fidelity by correcting the feature statistics at each denoising timestep.

### Quantitative evaluation

We quantitatively evaluated the performance in terms of appearance preservation and multiview consistency using preservation and consistency scores, respectively. Following prior image-generation evaluation practice, both metrics were computed using the cosine similarity between CLIP [58] image embeddings extracted by a pretrained CLIP image encoder. Because CLIP similarity primarily captures semantic and perceptual similarities instead of pixel-level alignment, we used it as a complementary perceptual metric along with qualitative comparisons. Let *f(·)* denote the CLIP image encoder and let all features be L2-normalized. The similarity between the two images $I_a$ and $I_b$ is computed as follows:

4 $$sim(I_a, I_b) = f(I_a)^\top f(I_b)$$

#### Preservation score

For each 3D model, let $$I^{ref} $$ be the reference image and $$I^{(v)}$$ be the rendered images of the textured mesh from *V* viewpoints. The preservation score measures how well the generated texture preserves the appearance of the reference image.

5 $$\mathrm{Preservation} = \frac{1}{V}\sum_{v=1}^{V} sim(I^{ref}, I^{(v)})$$

#### Consistency score

To measure the cross-view consistency, we compute the CLIP similarity between adjacent rendered views.

6 $$\mathrm{Consistency} = \frac{1}{V}\sum_{v=1}^{V} sim(I^{(v)}, I^{(v+1)})$$

where $I^{(V+1)}=I^{(1)}$. A higher score indicates better appearance consistency across neighboring viewpoints.

The preservation score measures the similarity between the input reference and rendered images, whereas the consistency score measures the similarity between adjacent renderings. The input mesh and its generated texture were projected onto eight viewpoints evenly distributed around the model, thus resulting in eight renderings. The results are listed in Table 1. Because of the attention controls in our appearance-control module, IP-ConTex achieved the highest preservation score. Paint3D achieved the highest multiview consistency because it generates content directly in the texture space. Among the methods based on RGB-space diffusion models, IP-ConTex achieved superior multiview consistency, which is attributed to its synchronized multiview diffusion and feature-synchronization strategies.

**Table 1:** Quantitative result

	TEXTure [7]	Paint3D [25]	PGC-3D [6]	IP-ConTex
Preservation score *↑ *	84.83%	82.69%	84.19%	89.25%
Consistency score * ↑ *	91.33%	91.84%	89.74%	91.62%

In addition to the visual comparisons in Fig. 6, we conducted a quantitative ablation study, as shown in Table 2. Overall, the full model achieved the optimum balance between appearance preservation and multiview consistency. Removing cross-attention control decreased both the preservation and consistency scores, thus confirming the advantage of image-prompt guidance in the early denoising stage for establishing a coherent global semantic layout and style across views. Moreover, removing feature synchronization reduced both metrics, thus demonstrating that synchronizing diffusion features is important not only for maintaining 3D consistency but also for preserving the reference appearance during cross-view propagation. The preservation score is a CLIP-based metric that primarily reflects high-level semantic and global visual similarities. Therefore, it does not necessarily increase monotonically with components that primarily improve the low-level color fidelity or fine-grained detail transfer. In particular, removing AdaIN yielded a slightly higher preservation score; however, this difference was marginal. Because AdaIN is primarily designed to align color statistics with the reference image, its benefit is more clearly reflected in perceptual color faithfulness, as shown in Fig. 6. Similarly, removing self-attention control yielded the highest consistency score but also the lowest preservation score. This suggests that without self-attention, the generated views become more homogeneous across viewpoints, which can increase the adjacent-view similarity while losing the fine-grained appearance details transferred from the reference image. By contrast, self-attention control encourages richer detail injection and geometry-aware appearance transfer, thus improving the visual fidelity of the final textures, although this effect is not fully captured by adjacent-view CLIP similarity alone. Collectively, these quantitative results are consistent with the visual ablations shown in Fig. 6: different components contribute to different aspects of texture quality, and the full model provides the most balanced performance overall.

**Table 2:** Quantitative ablation study

	Full model	w/o Cross-attention	w/o Self-attention	w/o Feature Sync	w/o AdaIN
Preservation score*↑*	89.25%	88.91%	87.19%	88.23%	89.30%
Consistency score*↑*	91.62%	90.49%	92.43%	91.32%	90.87%

### Time efficiency

As shown in Table 3, the proposed IP-ConTex effectively balances generation quality and computational efficiency. Compared with optimization- or fine-tuning-based methods, IP-ConTex does not require per-sample training. Specifically, TEXTure requires approximately 120 min per sample owing to the intensive DreamBooth fine-tuning process, whereas PGC-3D requires approximately 30 min for SDS-based optimization. By contrast, IP-ConTex completes the entire texture-generation process in approximately 4 min (approximately 263 s) on a single NVIDIA GeForce RTX 3090 GPU—comparable to the feedforward speed of Paint3D (approximately 3 min)—while maintaining higher detail consistency. Furthermore, IP-ConTex operates with a manageable peak GPU memory of 17.5 GB, thus rendering it accessible to standard consumer-grade hardware. To further analyze the computational overhead, we provide a detailed runtime breakdown in Table 4. The 50-step diffusion process is the primary bottleneck—accounting for 84.8% (223 s)of the total time—as it involves multiview synchronized denoising and the execution of the appearance-control module. The DDIM inversion and initialization phase requires 11.8% (31 s), whereas multiview back-projection and fusion in UV space are highly efficient, requiring only 9 s (3.4%) to aggregate the final texture. This efficiency demonstrates that our synchronization and control strategies add minimal overhead to the standard diffusion pipeline.

**Table 3:** Efficiency comparison

Method	Fine-tuning	Optimization	Time	Peak GPU memory
TEXTure [7]	Yes	No	Approximately 120 min	18.9 GB
Paint3D [25]	No	No	Approximately 3 min	12.3 GB
PGC-3D [6]	No	Yes	Approximately 30 min	20.1 GB
IP-ConTex	No	No	Approximately 4 min	17.5 GB

All measurements are obtained on a single NVIDIA GeForce RTX 3090 GPU

**Table 4:** Runtime breakdown of IP-ConTex

Stage	Time	Percentage
Initialization and DDIM inversion	31 s	11.8%
50-step diffusion	223 s	84.8%
Multiview back-projection and fusion	9 s	3.4%
Total	263 s	100%

## Discussion

### Superiority

IP-ConTex provides a promising solution for image-guided texture generation. Benefiting from the proposed appearance-control module, IP-ConTex can generate high-quality textures while preserving the appearance details of the reference image, thereby affording fine-grained control over the generation process. In terms of texture quality, IP-ConTex incorporates a synchronized diffusion module to ensure multiview consistency. Regarding detail preservation, the appearance-control module maintains detailed consistency between the generated textures and image prompts. Additionally, IP-ConTex is time efficient, as it does not require lengthy fine-tuning or optimization, thus enabling texture generation within minutes. Overall, the proposed approach reduces the cost and barriers to entry for 3D asset creation and facilitates the generation of high-quality, diverse textures for 3D models.

### Hyperparameter discussion

The three scheduling parameters $\tau_{sync}$, $\tau_{cross}$, and $\tau_{feat}$ control different denoising phases and exert intuitive effects on the generation behavior. $\tau_{sync}$ determines the duration for which synchronized multiview diffusion is applied; larger values generally improve global structural consistency across views, whereas excessively large values may reduce the flexibility of subsequent per-view refinement. $\tau_{cross}$ controls the duration of image-prompt guidance through cross-attention; increasing it strengthens the global appearance influence from the reference image, whereas overly large values may introduce appearance leakage that is less compatible with the target geometry. $\tau_{feat}$ determines the duration of diffusion feature synchronization; larger values improve the propagation of appearance details across views, whereas overly large values may overconstrain late-stage refinement. In our experiments, we used default parameter settings ($\tau_{sync}=0.6, \tau_{cross}=0.2, \tau_{feat}=0.8$) for all test cases and did not perform per-object tuning, thus suggesting that the method performs stably under a shared parameter setting across different object categories.

### Limitations and future studies

Although IP-ConTex performs well in generating consistent and high-quality textures, it presents certain limitations. First, undesired lighting information is embedded in the generated textures. For example, in Fig. 4, the heel of the second shoe model retains the highlight artifacts originating from the reference image, which is not conducive to downstream tasks such as relighting. Future studies can address this issue by designing novel textural representations that disentangle lighting from appearance. Second, the use of a fixed set of 11 predefined viewpoints may result in incomplete surface coverage, particularly for models with complex geometries and occluded regions. This may generate texture holes in insufficiently observed areas during generation. To mitigate this issue, an adaptive viewpoint sampling strategy can be developed to ensure greater surface coverage.

Another practical scenario is that multiple reference images from different viewpoints may be available. In the current implementation, we focused on a single-reference setting for a simple and clear analysis. Nevertheless, the proposed framework can be extended to this case by associating each reference image with its corresponding main view, followed by applying cross-image self-attention using a matched reference latent for that view. Subsequently, the appearance information from these reference-guided views can be propagated to the remaining views through shared self-attention and diffusion feature synchronization. Resolving inconsistent appearance cues across different reference images remains a challenge, as it requires techniques such as viewpoint-aware selection or confidence-weighted fusion. A systematic study of multireference guidance remains a promising direction for future research.

**Fig. 7:**
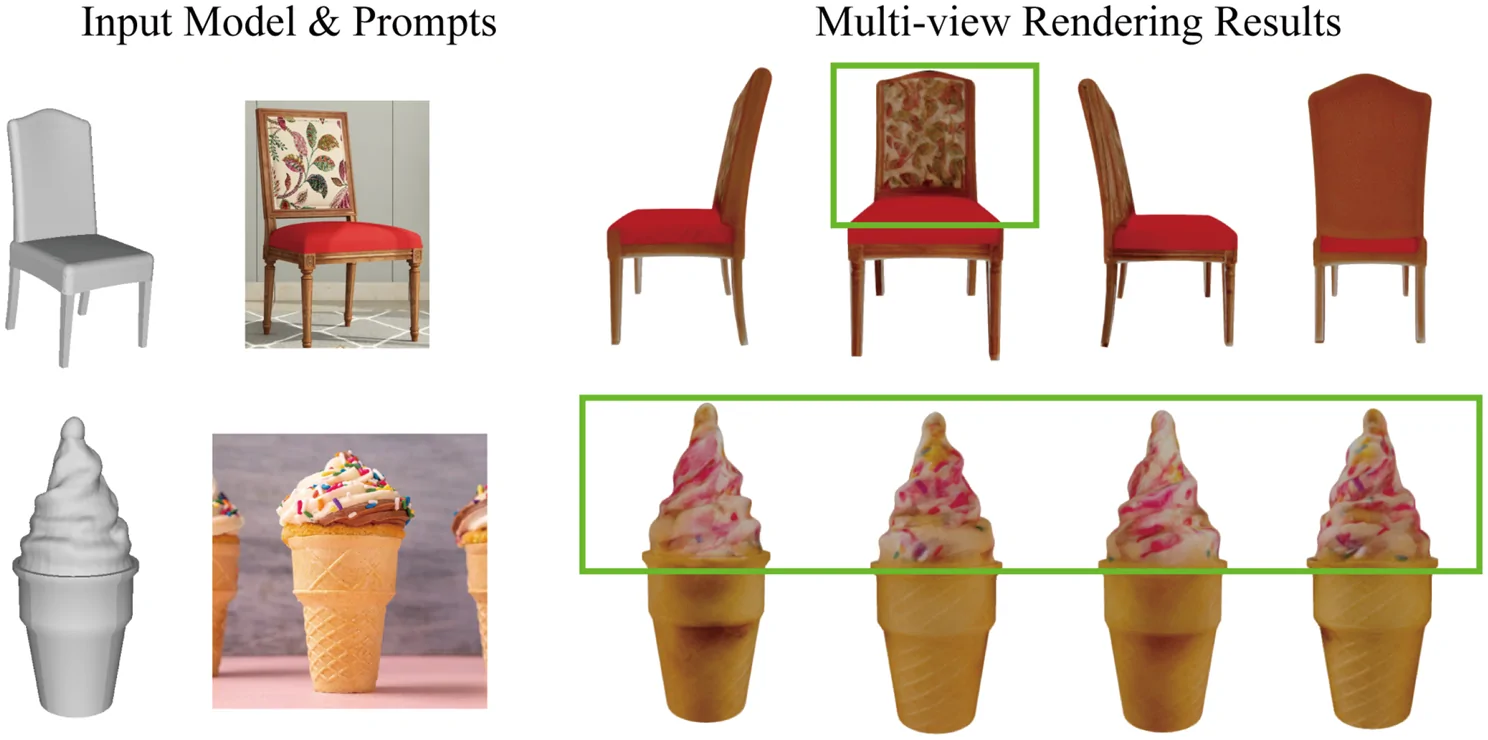
Failure case on high-frequency repetitive textures. When the reference image contains dense repeated patterns, the generated texture may appear oversmoothed or partially distorted after cross-view synchronization and fusion

Although IP-ConTex demonstrates excellent performance in preserving appearance consistency, it fails to effectively manage reference images containing high-frequency repetitive patterns, as illustrated in Fig. 7. Specifically, when the reference image contains dense stripes, checkerboard patterns, fine logos, or intricate embroidery, the generated textures may appear oversmoothed, while repetitive patterns may be attenuated during cross-view propagation and texture fusion. We attribute this limitation to the tradeoff between multiview consistency and high-frequency detail preservation. As the proposed framework repeatedly synchronizes and fuses information across views to maintain 3D consistency, minor discrepancies in the pattern phase, alignment, or sampling between different views may accumulate, thus resulting in an averaging effect in the fused texture space. Consequently, while the overall structure and style remain coherent, extremely fine repetitive details are not consistently preserved. A promising direction for future work is to incorporate detail-aware refinement mechanisms, such as patch-level appearance transfer, frequency-aware fusion, or texture super-resolution modules, to better preserve high-frequency repetitive details while maintaining cross-view consistency.

## Conclusions

In this study, we proposed IP-ConTex, a novel method for detail-consistent texture generation guided by image prompts. By leveraging a pretrained 2D image diffusion model, IP-ConTex generates high-quality textures that preserve the appearance details of a reference image for a specified 3D geometric model. The proposed method comprises two key modules: synchronized multiview diffusion and appearance control. The former ensures structural and layout consistency across multiple views by sharing denoised content through a latent-space texture map at each timestep. The latter injects appearance information into the generation process and propagates it across views using cross-attention control, self-attention control, diffusion feature synchronization, and a color-adjustment strategy. Experimental results demonstrate that IP-ConTex effectively preserves fine-grained appearance details and achieves superior texture quality compared with existing state-of-the-art methods.

## Data Availability

The 3D models used in this work are publicly available and can be accessed from the Objaverse dataset at https://objaverse.allenai.org/.

## Abbreviations

2D: Two-dimensional
3D: Three-dimensional
AdaIN: Adaptive instance normalization
SDS: Score distillation sampling
VSD: Variational score distillation
